# The Role of Intracoronary Imaging for the Management of Calcified Lesions

**DOI:** 10.3390/jcm12144622

**Published:** 2023-07-11

**Authors:** Stylianos Petousis, Emmanouil Skalidis, Evangelos Zacharis, George Kochiadakis, Michalis Hamilos

**Affiliations:** Cardiology Department, University Hospital of Heraklion, Voutes and Stavrakia, 71110 Heraklion, Crete, Greece; skalides@med.uoc.gr (E.S.); kochiadg@gmail.com (G.K.)

**Keywords:** calcified coronary lesions, IVUS, OCT, PTCA

## Abstract

Interventional cardiologists in everyday practice are often confronted with calcified coronary lesions indicated for percutaneous transluminal coronary angioplasty (PTCA). PTCA of calcified lesions is associated with diverse technical challenges resulting in suboptimal coronary stenting and adverse long-term clinical outcomes. Angiography itself offers limited information regarding coronary calcification, and the adjuvant use of intracoronary imaging such as intravascular ultrasound (IVUS) and Optical Coherence Tomography (OCT) can guide the treatment of calcified coronary lesions, optimizing the different stages of the procedure. This review offers a description of why, when, and how to use intracoronary imaging for PTCA of calcified coronary lesions in order to obtain the most favorable results. We used the PubMed and Google Scholar databases to search for relevant articles. Keywords were calcified coronary lesions, intracoronary imaging, IVUS, OCT, coronary calcium modification techniques, PTCA, and artificial intelligence in intracoronary imaging. A total of 192 articles were identified. Ninety-one were excluded because of repetitive or non-important information.

## 1. Introduction

Coronary calcification often coexists with atheromatous disease, sharing common predisposing factors such as arterial hypertension, diabetes mellitus, dyslipidemia, and smoking. Considering the aging of the general population and the increase in diseases such as diabetes mellitus and chronic renal failure, the prevalence of coronary artery calcification is expected to increase and become an even more frequent finding in catheterization laboratories. Percutaneous transluminal coronary angioplasty (PTCA) is a common therapeutic intervention, particularly in developed countries. Diverse anatomical and pathophysiological parameters can hinder the procedure, and coronary calcification certainly represents one of the major causes of short- and long-term negative outcomes. Moderate to severe coronary calcification is a common finding in lesions undergoing PTCA, varying from 18% to 30%, and poses significant technical difficulties in stent placement [1,2,3]. Inadequate calcified plaque preparation can lead to stent underexpansion, which is strongly correlated with stent thrombosis, stent restenosis, and target lesion failure [4,5,6]. Intracoronary imaging can provide diverse information about the extent and morphology of coronary calcification, aiding in the choice of the most adequate calcium modification strategy, evaluating its effect, and optimizing coronary stenting results.

Coronary calcification is strongly associated with male sex, Caucasian race, advanced age, and comorbidities such as diabetes mellitus, arterial hypertension, chronic kidney disease, dyslipidemia and smoking [7,8]. Most importantly, coronary artery calcification has a well-documented correlation with atheromatic plaque burden and the extent and complexity of atherosclerotic coronary artery disease (multivessel disease, multiple lesions, chronic total occlusions, and bifurcation lesions) [8,9]. This association has an interesting historical reference, as it appears in a manuscript of 1799, written by two physicians at the time [10].

## 2. Pathophysiology of Coronary Calcification

The mechanisms underlying coronary calcification are not fully understood. Coronary calcification follows the development of atherosclerotic plaque, and it seems that calcium phosphate deposits at sites of accumulation of debris derived from the apoptosis of smooth muscle cells. This is the main initial step of microcalcification formation [11] and macrophages have also a pivotal role [12]. Microcalcifications coalesce over time in larger formations, resulting in calcium speckles, fragments, sheets, plates, and calcified plaques [1]. Other mechanisms of the calcification process include the transformation of smooth muscle cells into osteoblast-like phenotypes under the effect of stress factors (inflammation, hypoxia, and flow disturbances), the loss of systematic calcification inhibitors, and disturbances in calcium-phosphate metabolic equilibrium [13,14]. Calcification alters the composition and architecture of vessels. The vessel becomes less compliant, and its mechanical and physiological properties are significantly affected [15]. Different coronary calcification patterns are observed. Calcium can be superficial in an intimal-luminal location, deeper in a medial adventitial position, or both. Spotty calcifications mostly correlate with unstable atherosclerotic plaques and acute coronary syndromes [16,17]. Nodular calcification, a particular form of calcification, is derived from the gradual fracture of calcium sheets, extending towards the vessel media or lumen, and is associated with acute coronary events, often causing fibrous cap rupture [1,18].

## 3. Clinical Implications

Coronary calcification is associated with several unfavorable issues during percutaneous treatment of coronary lesions. It causes difficulty in balloon crossability and dilation, but also in stent expansion, resulting in time-consuming interventions with excessive use of contrast and radiation. Severe complications can occur, such as balloon rupture, stent loss distortion or fracture, and sometimes coronary dissection or perforation because of the disparate forces applied on the coronary vessel wall from balloon dilation, impeded by calcium [2,8,19,20]. Damage to the stent polymer and altered drug kinetics have also been described [21]. Calcific plaques hamper adequate lesion preparation, predisposing to stent underexpansion and malapposition [22]. There is strong evidence that patients with calcified coronary stenosis who undergo PTCA have a worse prognosis with high percentages of major adverse cardiac events (MACEs), restenosis, and target lesion revascularization (TLR) [23,24,25] (Table 1).

Data from two large randomized controlled trials (ACUITY and HORIZONS-AMI) derived from patients with acute coronary syndromes who underwent PTCA showed that moderate-to-severe lesion calcification was a frequent finding (26.1% and 5.9%, respectively). These patients had a higher risk of stent thrombosis and TLR at one year [8]. Bourantas et al., in a metanalysis of seven clinical trials, reported that severe calcification was detected in 20% of patients. These patients underwent complete revascularization less frequently (48% vs. 55.6%) and had an increased mortality rate [2]. This finding was recently confirmed in a substudy of the SYNTAXES trial, which showed that heavy calcification in revascularized patients either with PTCA or CABG was associated with an increased mortality rate at 10 years [26]. Généreux et al. found that coronary artery calcification in patients who underwent PTCA was very common (30.8%) and strongly correlated with MACEs and bleeding complications [27]. Huisman et al., after analyzing the TWENTE and DUTCH PEERS trials, concluded that severe lesion calcification in patients with stable coronary disease which underwent PTCA was correlated with an increased risk of MACE at one year (target vessel failure 16.4% vs. 9.8%, cardiac death 4.4% vs. 1.5%, target vessel myocardial infarction 7.6% vs. 3.4%, and stent thrombosis 1.8% vs. 0.4%) [28]. Similarly, patients with acute coronary syndrome and severe coronary lesion calcification treated with PTCA showed high rates of target vessel revascularization during the years of the follow up period [29]. Copeland-Halperin et al. reported that, in a large cohort of patients who underwent PTCA with drug-eluting stents, moderate and severe calcification was associated with high rates of MACE at one year (death 5.5% for severely calcified lesions, 3.3% for moderate and 1.8% for mild/noncalcified lesions) [3]. Jinnouchi et al. found that in patients with heavy coronary calcification, treated with IVUS-guided PCI and rotational atherectomy, TLR at one year was significantly higher when calcified nodules were present [30]. In a pooled analysis of the ISAR-TEST 4 and 5 trials, MACE rates at 10 years follow up among patients who underwent PTCA proportionally increased according to the degree of calcification of the lesion’s calcification [31]. All of these studies consistently showed the negative impact of calcium in the coronaries on hard clinical outcomes.

**Table 1:** Clinical studies regarding patients with calcified lesions treated with PCI.

Author	Population	Main Results
Onuma Y et al. *Catheter Cardiovasc Interv.* 2010 [24]	212 patients, 68 with moderately or severely calcified lesions	MACE rates in patients treated with everolimus eluting stents for calcified lesions were higher than in those for non-calcified lesions
Bourantas et al. *Heart* 2014 [2]	Data from 7 clinical trials, 6296 patients undergoing PCI, 20% of them had severe calcification	Patients with severe calcification undergoing PCI are less likely to receive complete revascularization and have a worse prognosis with increased mortality
Généreux P et al. *JACC* 2014 [8]	Data from 6855 patients with ACS who underwent PCI, pooled from 2 large-scale randomized controlled trials (ACUITY and HORIZONS-AMI)	Moderate/severe lesion calcification was frequent in patients with NSTEMI and STEMI and it was strongly predictive of stent thrombosis and ischemic Target Lesion Revascularization at 1 year
Copeland-Halperin RS *Catheter Cardiovasc Interv.* 2018 [3]	Retrospective analysis of a large, multiethnic cohort of patients (12.445) undergoing PCI with new generation DES between 2009 and 2013	Moderate to severe calcification was found in 10% and 8% of patients respectively, independently associated with adverse outcomes
Huisman J et al. *Am Heart J.* 2016 [28]	1423 patients with stable angina/342 with severe calcification (analysis of the TWENTE and DUTCH PEERS randomized trials)	Severe calcification increases the risk of adverse cardiovascular events in patients with stable angina treated with newer generation DES
Kawashima H *JACC Cardiovasc Interv.* 2022 [26]	Substudy of the SYNTAXES study	At 10 years, the presence of a heavily calcified lesion was an independent predictor of mortality, with a similar prognosis following PCI or CABG
Huisman J et al. *Journ of Cardiol* 2017 [28]	1779 ACS patients, 340 with severe target lesion calcification (metanalysis of the TWENTE and DUTCH PEERS randomized trials)	Patients with severe calcification had a higher Target Vessel Revascularization rate
Guedeney P et al. *JACC Cardiov Interv* 2020 [25]	Patient’s (19,833) data, pooled from 18 randomized trials evaluating DES; 6211 presented moderate or severe coronary calcification in angiography	PCI of moderate or severe coronary calcified lesions was associated with adverse outcomes at 5 years
Rheude T et al. *Eurointervention* 2023 [31]	4953 patients with 6924 lesions included (analysis of the ISAR-TEST 4 and 5 randomised trials), moderate and severe calcification presented in 25.8% and 8.0%, respectively	At 10 years after PCI with new-gen DES, there was an increase in adverse events by severity of coronary calcification, independent of the different polymer-coating
Jinnouchi, H et al. *J Atheroscl and Thromb* 2022 [30]	249 calcified lesions underwent intravascular ultrasound-guided PCI with rotational atherectomy (RA), 100 of them presented calcified nodules	In heavily calcified lesions treated with RA before PCI, calcified nodule was associated with high target lesion revascularization rate at one year

Abbreviations: MACE: Major Adverse Cardiac Events; NSTEMI: Non ST segment Elevation Myocardial Infarction; STEMI: ST segment Elevation Myocardial Infarction; PCI: Percutaneous Coronary Intervention; CABG: Coronary Artery Bypass Graft; DES: Drug Eluting Stents; RA: Rotational Atherectomy.

## 4. Imaging of Coronary Calcified Lesions

Coronary angiography has low sensitivity but high specificity for calcium detection. Calcifications appear as dark linear formations along the contour of the coronary artery during fluoroscopy. Coronary calcification can be mild when no radiopacities are visible on coronary angiography, or moderate when radiopacities can be detected during cardiac cycle motions and before contrast medium injection. In cases of severe calcification, these formations can be identified in both borders of the vessel wall before contrast injection, while the vessel lumen can have a “foggy’’ appearance on angiography. Despite its high specificity, angiography alone offers only a limited assessment of calcified lesions, as it cannot provide complete information regarding calcium’s depth, circumferential extent (arc) and precise lesion length.

**Figure 1:**
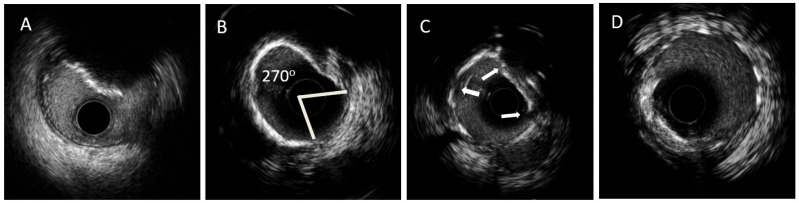
IVUS images during PTCA of calcified lesions. Legend: (**A**). Calcified nodule. (**Β**). Concentric calcium of 270 degrees. (**C**). Presence of calcium fractures after IVL treatment (white arrows). (**D**). IVUS after stent implantation. Abbreviations. IVUS: IntraVascular UltraSound, PTCA: Percutaneous Transluminal Coronary Angioplasty.

Intracoronary imaging using IVUS and/or OCT (Table 2) can provide a more accurate estimation of calcified lesions, helping the operator choose appropriate calcium modification tools and guide stenting when PTCA is indicated. IVUS uses highly penetrable ultrasound waves, giving operators sequential cross-sectional images of the coronary vessel, permitting vessel, lumen, and lesion length measurements. Conventional IVUS systems have a spatial resolution of approximately 100 μm and a tissue depth penetration of 4–8 mm. Newer systems of 45 MHZ (Refinity, Philips) or even 60 MHz (Opticross HD, Boston Scientific, Acist HDi IVUS system) offer a better spatial resolution of up to 22 μm. In studies examining post-mortem coronary histologic findings, IVUS has high sensitivity and specificity for detecting calcified lesions [32]. The diagnostic value of IVUS compared to coronary angiography alone was highlighted in the pioneering work of Mintz et al. [33], which was confirmed in later studies [34]. However, IVUS has lower sensitivity in the presence of microcalcifications (because of its relatively low resolution) and fibrous plaques [35,36]. Calcium typically appears as hyperechoic bright areas with acoustic “shadowing’’ as calcium reflects echo waves, making unfeasible deeper imaging of the calcified lesion or vessel’s wall, especially in cases of heavily calcified plaques. This phenomenon impedes the use of IVUS to properly estimate calcium thickness. An indirect method for thickness evaluation is the presence of posterior reverberations which indicate a calcium thickness of <0.5 mm, while a complete echo shadowing indicates a thicker lesion of >1 mm [37]. IVUS detects deep calcium (into the deep media and adventitia) owing to its ultrasound properties. Calcified lesion location can be characterized as superficial when its outer margin is positioned close to the intima-lumen interface, deep when it is found deeper in the media-adventitia, or both when it occupies all vessel layers. IVUS can provide an accurate estimation of calcium circumferential extension (arc) and length. Calcified nodules can be identified with IVUS as convex or irregular formations, with severe acoustic “shadowing”, often protruding into the lumen of the vessel [37] (relative IVUS images are shown in Figure 1).

**Figure 2:**
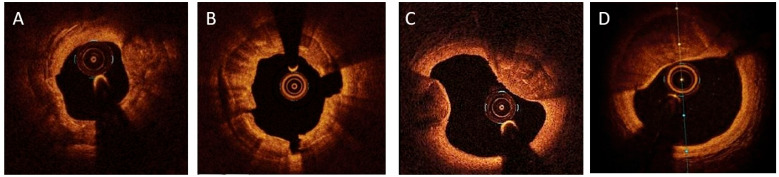
OCT images of coronary calcified lesions. Legend: (**A**). Concentric calcium appearance. (**B**). Calcium fractures. (**C**). Calcified nodules. (**D**). Eccentric calcium appearance. Abbreviation: OCT: Optical Coherence Tomography (Images are courtesy of Abbott, Diegem, Belgium).

OCT is based on infrared spectrum light to obtain high-resolution images of 10–20 μm but with a limited depth of tissue penetration of 1–2 mm. Although OCT is much more sensitive than angiography, it is less sensitive than IVUS in detecting calcified lesions [38]. In contrast, infrared light penetrates calcium, allowing visualization beyond the arterial wall. Calcium appears as low-intensity signal areas with well-delineated borders, which permits accurate measurements of calcium area, thickness, and volume, in addition to its circumferential extension (arc) and length (OCT images are shown in Figure 2). Moreover, it has a better ability to detect microcalcifications than IVUS because of its much better resolution capability [39,40,41]. There are some limitations of OCT. The low penetration properties of OCT limit the estimation of deep calcium, whereas the interposition of lipid or necrotic content attenuates light penetration, rendering calcium evaluation inaccurate [41]. In addition, OCT cannot be used in large-diameter vessels (owing to its limited penetration depth) and in the presence of aortic ostial lesions, as it presents technical limitations [42], where IVUS has an obvious advantage [43]. Calcified nodules appear as nodular formations of various dimensions and locations [44], whereas disruption of the fibrous cap is correlated with acute coronary syndromes and cardiac death [45,46]. OCT offers the unique potential of distinguishing between the two main morphologies of calcified nodules: eruptive and protruding [47,48]. The eruptive form has an irregular surface appearance, often coexisting with disruption of the intimal fibrous cap and presence of thrombus. This pattern of calcified nodules, despite better stent expansion results than the protruding form, is correlated with worse clinical outcomes in terms of cardiac death and target lesion failure [47,48]. On the other hand, the protruding form is associated more often with stent underexpansion, as it affects vessel compliance [47].

Recently, the FDA approved high-frequency (HF) OCT (Gentuity, Nipro Corporation, Osaka, Japan). HF-OCT uses a lower profile catheter (60% compared to conventional OCT), resulting in better crossability through tightly calcified lesions. Moreover, it is capable of achieving a faster pullback registration. Both IVUS and OCT, in addition to evaluating vessel diameter, lesion length, and lumen area, can provide important information related to calcified lesion characteristics, guiding lesion preparation, stent implantation, and assessment of post-PTCA results. Because both IVUS and OCT imaging technologies have strengths and weaknesses, new hybrid systems combining these two methods have been developed (Novasight Hybrid System, Conavi Medical Inc., Toronto, ON, Canada and Dual Sensor system, TERUMO, Tokyo, Japan). Although coronary imaging with near-infrared spectroscopy (NIRS) is used to estimate the intraplaque lipid content, hybrid forms of IVUS/NIRS and OCT/NIRS can contribute to global plaque characterization, identification of vulnerable plaques, and optimization of stenting [49].

Earlier IVUS studies showed that coronary calcification impedes stent expansion, particularly when calcium is eccentric and thick [50], suggesting the need for lesion modification. Even after inflation of balloons at high pressures, moderate to severe calcified lesions estimated with IVUS negatively affect stent expansion in proportion to the calcium arc [51]. Similar problems with stent expansion were observed when OCT was used, as reported by Kobayashi et al. [22]. The extent of calcium has a negative impact on optimal stent expansion, and accordingly, on PCI success. Interventional cardiologists must consider diverse parameters of calcified lesions, such as calcium arc, area, thickness, length, and location, to perform an uncomplicated, successful PTCA. There is evidence that the presence of a larger arc and smaller thickness of calcium, estimated by OCT, increases the probability of fracture formation after the application of rotational atherectomy and/or balloon inflation [52,53]. Fujino et al. developed an OCT-based calcium scoring system, analyzing parameters from a retrospective study to predict stent underexpansion [54]. It was found that calcified lesions with a score of 4 (maximum calcium arc of >1800 (2 points), length > 5 mm (1 point), and maximum thickness of >0.5 mm (1 point), a score of 4 points predicted stent underexpansion.

A similar IVUS-based scoring system that identifies calcified lesions at risk for stent underexpansion was proposed by Zhang et al. based on a retrospective observational study [55]. In their score calculation, they included 360° of superficial calcium (1 point), superficial calcium arc >270° longer than 5 mm (1 point), presence of a calcified nodule (1 point), and vessel diameter smaller than 3.5 mm (1 point). Calcified lesions with a score ≥2 have an increased risk of stent underexpansion. It seems that when an IVUS-guided PCI strategy is adopted in lesions with moderate-to-severe calcification, it is associated with favorable clinical outcomes, similar to PCI in lesions with no or mild calcification [56]. Finally, calcified lesions amenable to PTCA in which calcium cannot be detected by angiography and are visible only with IVUS/OCT have a low risk of stent underexpansion [37].

Detailed estimation and stratification of calcium using intravascular imaging helps to select the most appropriate calcium modification tools and even evaluate their effects.

## 5. Tools and Techniques for Calcified Coronary Lesions Treatment

### 5.1. Non-Compliant Balloons

Non-compliant balloons are used very often and are more effective and safer than semi-compliant balloons, as they dilate lesions more uniformly and at higher inflation pressures. They can be used successfully in mild or mild to moderate calcified lesions, while in severe calcification, they dilate the lesion asymmetrically at high pressures, causing complications (balloon rupture, coronary dissection, or perforation) [57]. Nevertheless, they should be used after lithotripsy balloon or rotational atherectomy to optimize and confirm appropriate lesion preparation before stent implantation [58].

### 5.2. Super High-Pressure Balloons

The OPN high-pressure balloon (SIS Medical AG, Winterthur, Switzerland) has a structure of two layers of non-compliant balloons with an inflation capacity of 30 to 45 atm, without significant increases in balloon diameter. This balloon can be effective in eccentric and concentric calcified lesions, particularly in undilatable lesions. Attention must be paid to the presence of calcified nodules where vessel rupture can occur after balloon inflation at very high pressures. Additionally, the OPN balloon can be used for post-dilatation for stent underexpansion when conventional non-compliant balloons fail to achieve adequate expansions [59].

### 5.3. Cutting Balloons

Cutting balloons have microblades placed on their surfaces, causing controlled longitudinal incisions during inflation. Thus, they can modify fibrous or calcific plaques. Their use is limited because of their difficult crossability and deliverability; however, this has improved in the latest edition. They appear to be more effective for calcium modification than scoring balloons when intravascular imaging is used [60].

### 5.4. Scoring Balloons

Scoring balloons have a better crossability and deliverability profile than cutting balloons, causing less vessel wall trauma and lower rates of coronary dissections. They apply focal, uniform forces to the atherosclerotic plaque surface through scoring elements mounted on their surface. Some small studies have shown promising results of scoring balloons on their efficacy in calcified lesions [61,62,63] and more results from ongoing studies are expected.

### 5.5. Lithotripsy Balloon

Intracoronary lithotripsy (Shockwave Medical, Santa Clara, CA, USA) uses a semi-compliant balloon advanced over a workhorse support guidewire containing small emitters that generate sparks that produce expanding vapor bubbles in the balloon, resulting in bursts of acoustic waves that crack both superficial and deep calcium. This therapy is very effective for both superficial and deep calcium, has no effect on soft tissues, and ameliorates vessel compliance [64]. Subsequent dilation of the calcified lesion with a non-compliant balloon is often performed before stent implantation [65]. With this method, inflation of balloons at very high pressures can be avoided by sparing vessels from traumatic lesions. Intracoronary lithotripsy is effective in both eccentric and concentric calcified lesions and calcified nodules [66,67], which have been previously identified with intravascular imaging. Lithotripsy balloons are commonly used to treat stent underexpansion in calcified lesions [68,69,70] when balloon-based strategies usually fail. Recent studies have shown that this method can be used safely with optimal results [71,72,73], although long-term clinical data are missing, and concerns about damage to stent polymers exist [74]. In most of the cases, the use of intravascular imaging for calcium evaluation before stent implantation could probably have prevented the use of intravascular lithotripsy as a “bail out’’ therapy for stent underexpansion.

### 5.6. Rotational Atherectomy

Rotational atherectomy is an effective tool for the treatment of severely calcified coronary lesions with PTCA. This technology (Boston Scientific, Marlborough, Massachusets) uses a diamond-covered burr advanced on a specialized wire of 0.009 inch (Rotawire Floppy or Extra support), rotating at very high speeds of 140,000 to 200,000 rpm, resulting in ablation of the calcified and fibrotic material of the atherosclerotic plaque, sparing soft tissue. In this way, the lumen diameter of the vessel increases and becomes smoother, while calcium cracks or “fissures’’ can be detected using intravascular imaging [75,76]. This allows for more effective balloon inflation and successful stent placement. The technique is accompanied by a higher incidence of complications compared to conventional PTCA, including coronary dissection, vessel perforation, slow-flow/no-reflow phenomenon, atrioventricular block, rotawire fracture, and burr entrapment [77]. IVUS findings such as longer lesion length, maximum number of reverberations, and greater arc of calcification at the MLA may predict slow flow after RA [78]. There is evidence that OCT-guided rotational atherectomy results in better stent expansion than IVUS-guided rotational atherectomy [79,80]. Rotational atherectomy is preferred in cases of balloon uncrossable, serial, or long calcified lesions ideally with superficial and concentric pattern of calcium in intracoronary imaging.

### 5.7. Orbital Atherectomy

This new tool for the treatment of severely calcified lesions (Cardiovascular Systems, Inc. Diamond 360 Minnesota YSA) consists of a single-sized, full diamond coated crown of 1.25 mm that is mounted eccentrically, rotating at high speeds (80.000 or 120.000 rpm mode) in an elliptical trajectory, selectively ablating rigid calcified tissue. Compared with rotational atherectomy, the depth of ablation increases with the rotational speed of the crown. Moreover, it acts on rigid tissue in both forward and backward directions, reducing crown entrapment [81,82]. Intravascular imaging using OCT reveals deeper and more extensive modification of calcified lesions, resulting in better stent apposition [76] in comparison with rotational atherectomy.

### 5.8. Excimer Laser Atherectomy

Excimer Laser Coronary Atherectomy (ELCA) uses pulses of high-energy ultraviolet light, and its action is mediated by the synergistic effects of photochemical, photomechanical, and photothermal mechanisms [83,84]. This method can be applied in combination with rotational atherectomy (RAZER technique) [85]. Although there is some positive evidence for the effectiveness of this technology [86,87,88], data on its use are limited. It must be noted that the presence of severe calcification limits its efficacy [89], and most importantly, the availability of the method is limited.

## 6. Use of Imaging to Guide the Treatment of Calcified Lesions

It is widely accepted that intravascular imaging using IVUS or OCT plays a pivotal role in the assessment of calcified coronary lesions and identification of high-risk characteristics for stent underexpansion [40,43,90]. Despite the existence of position papers and a recent consensus document [58,91,92], there are no dedicated guidelines regarding the guidance of calcium modification tools according to the intravascular imaging findings. Diagnostic and therapeutic approaches depend mainly on the availability of the devices and local expertise. Published knowledge, diverse proposed algorithms, and shared experience provide useful information regarding the appropriate use of intravascular imaging for calcium management.

In cases of angiographically mild-to-moderate calcification, the lesion can be initially prepared with non-compliant balloon inflations, ensuring its full expansion in at least two different projections. If the non-compliant balloon is not fully expanded or a “waist’’ phenomenon is noted, intravascular imaging with IVUS or OCT can be considered to further estimate calcium [92] (as it may have been underestimated) or directly escalate the calcium modification technique. If the aforementioned high-risk criteria are not met on intracoronary imaging, further lesion preparation using cutting, scoring, or super-high-pressure balloons can be performed. Generally, balloon-based techniques can adequately treat mild-to-moderately calcified coronary lesions. Super high-pressure balloons can be effective when NC balloons fail and in the case of underexpanded stents.

In cases of angiographically moderate to severe calcification or ambiguous appearance, intravascular imaging can be of crucial importance [43,56,90,91,92] before any intervention to accurately assess calcium length, arc, and depth. OCT has an obvious advantage over IVUS for accurate estimation of calcium thickness and volume. If high-risk characteristics are detected with either IVUS or OCT, lesion modification using more aggressive techniques such as rotational/orbital atherectomy or intravascular lithotripsy is obligatory. Furthermore, intravascular imaging can help choose the most adequate burr size and IVL balloon diameter according to vessel dimensions and provide information regarding stent size and length prior to implantation. Mechanical atherectomy strategies are mostly used in balloon uncrossable stenoses, in long diffuse calcified lesions, in the presence of calcified nodules, or when calcium distribution is eccentric. Although rotational atherectomy effectively ablates superficial calcium, it has no effect on deep calcium. In such cases, rotational atherectomy can be used in combination with intravascular lithotripsy if there are few focused locations of deep calcium along a long lesion. Orbital atherectomy can offer advantages for long lesions with concomitant superficial and deep calcium localizations, as it can provide a more profound ablation effect, increasing the rotational speed of the crown. Furthermore, the same crown can be used equally for coronary segments of different diameters. In cases of tortuous vessels or steep angulations, atherectomy should be avoided as it can cause severe complications (wire fracture, burr entrapment, coronary dissection, and perforation). IVL is preferred in cases of focused calcified lesions, preferably those with concentric and deep calcium distributions. IVL is also effective in large-diameter vessels and bifurcation lesions, particularly in the presence of a large side branch when keeping a second guidewire in the jeopardized side branch is of critical importance. An emerging, although off-label use of IVL is stent underexpansion due to underlying inadequately modified calcium, a situation which could be probably avoided if proper use of intracoronary imaging had been previously performed.

**Figure 3:**
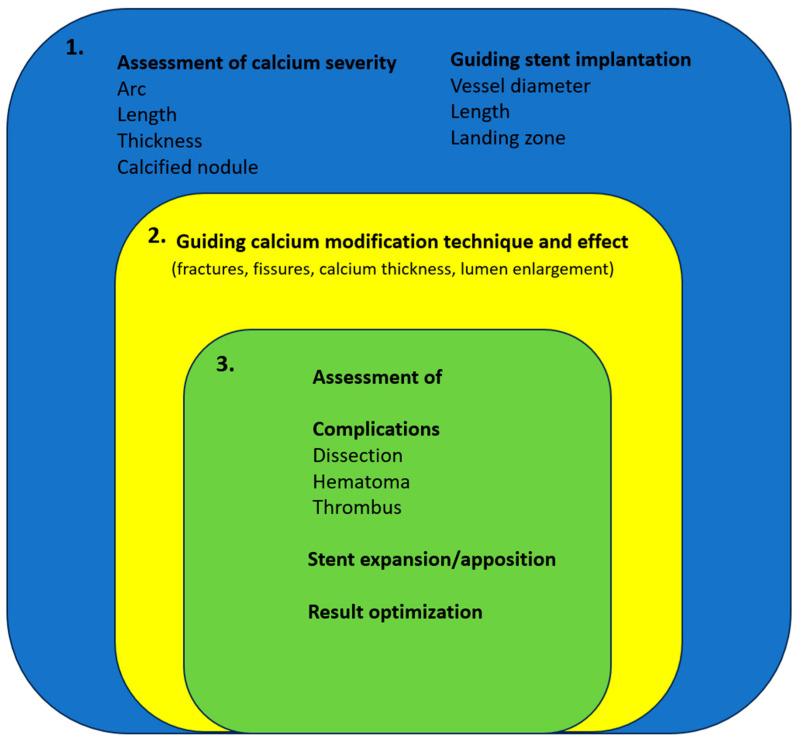
Use of intracoronary imaging for the treatment of PCI indicated calcified lesions.

**Figure 4:**
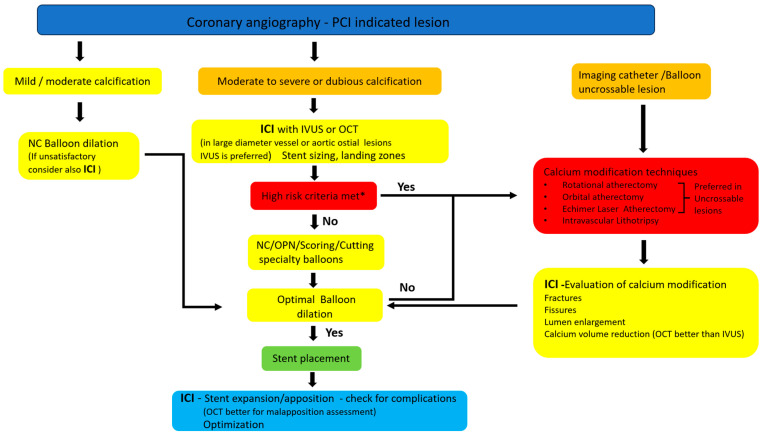
Algorithm of the use of intracoronary imaging in calcified lesions with indication for PCI. Abbreviations: PCI: Percutaneous coronary intervention, ICI: Intracoronary imaging, IVUS: IntraVascular UltraSound, OCT: Optical Coherence Tomography, NC: Non-compliant. Leggend: Asterisk * refers to intracoronary imaging high risk criteria as follows: IVUS high risk criteria: 1. Superficial calcium of >270° (longer than 5 mm), 2. 360° superficial calcium, 3. Calcified nodule, 4. Vessel diameter ≤ 3.5 mm, OCT high risk criteria: 1. Calcium max angle > 180°, 2. Calcium max thickness > 0.5 mm, 3. Calcium length > 5 mm.

After applying the selected calcium modification technique, intravascular imaging can provide information on its effectiveness. Calcium fractures and luminal enlargement indicate better vessel compliance and predict adequate stent expansion [52,93]. If the result obtained is unsatisfactory, an additional modifying technique should be considered (e.g., adding IVL in focused lesions with thick calcium after RA). After stent placement, intracoronary imaging can provide additional data, such as geographical miss, stent expansion, apposition, and minimum stent cross-sectional area. Complications, such as dissection, intramural hematoma, thrombus, and stent fracture, can also be identified using intravascular imaging. Such complications are often observed, particularly after a complex treatment strategy is used for calcified lesions. The use of intracoronary imaging for the treatment of calcified lesions is shown in Figure 3 and a relative algorithm is shown in Figure 4.

## 7. Future Implications

Data on the use of intracoronary imaging for the choice of the most appropriate calcium modification tool for guiding PTCA are scarce, the field is rapidly evolving, and related studies are needed. In most cases, the use of conventional balloons is the default practice, whereas ablation techniques and lithotripsy are used as bailout therapy. One reason for this is the high cost of intracoronary imaging and modification tools. On the other hand, the impact of underexpanded stents and periprocedural complications due to inadequate calcium assessment and treatment also increases the cost of treatment for such complex lesions.

Coronary calcification can have variable patterns and its arc, depth, and thickness are not always the same. The choice of the most suitable modification technique is difficult, and the effect that it will provide is not always predictable. Recently, a series of studies on the use of artificial intelligence in the field of intracoronary imaging have emerged. Deep learning algorithms use convolutional neural networks specifically designed and trained to analyze a large number of images. This allows the evaluation of large amounts of data derived from IVUS or OCT for different vessel segments in a few seconds. In the evolving field of artificial intelligence, several deep learning models for intracoronary imaging have already been developed. Such models can provide a plethora of information, helping the operator in different stages of the PTCA. Cho H. et al. developed an IVUS-based deep learning algorithm for quick and accurate coronary plaque characterization, oriented to the identification of high-risk plaque features, quantifying both calcium extent and fibroatheroma [94]. A promising IVUS-based deep learning method was proposed by Nishi et al., which provides automatic measurements of the lumen vessel and stent area, facilitating stent implantation and optimization [95]. Min et al. developed an IVUS deep learning system that elaborates data as stent length, diameter and dilation pressure, balloon diameter and max inflation pressure, predicting preprocedural stent underexpansion with high accuracy (94%) [96]. Other researchers have proposed OCT-based, automated, deep learning combined algorithms for atheromatic plaque characterization by detecting fibrolipidic and fibrocalcific components [97,98]. Accordingly, appropriately trained convolutional neural networks, by obtaining multiple OCT images, can automatically detect specific characteristics of calcified lesions (length, arc, and thickness) by obtaining multiple OCT images [99]. Similar methods of artificial intelligence after analyses of OCT images can provide calcium risk scores, facilitating the modification strategy of the lesion and stent placement [100], which can predict stent underexpansion and malapposition in calcified lesions undergoing PTCA [101].

In the future, evolved forms of intravascular imaging will be used based on IVUS and OCT technologies, probably in hybrid forms, with higher resolution properties and lower profiles. Artificial intelligence will accurately indicate the most appropriate calcium modification technique or a combination of these, guiding stent implantation and predicting PTCA results. A synergistic integration of clinical information (patient characteristics, comorbidities, and clinical syndromes) elaborated by specialized deep learning algorithms will provide an individualized estimation of the patient’s clinical outcome.

## 8. Conclusions

The number of patients with calcified coronary lesions is expected to grow in the future due to the aging of the general population and the increase in relative comorbidities in developed countries. PTCA in such lesions is associated with high rates of peri-interventional complications and procedural failures. Intravascular imaging using IVUS or OCT can be used pre-procedurally, indicating the most adequate calcium modification techniques and guiding stent implantation, peri-procedurally evaluating the modification result, and post-stent deployment to optimize the implantation and check for complications. More studies are needed in this field, while newer devices, techniques, and artificial intelligence tools are expected to play pivotal roles in the effective and uncomplicated treatment of calcified lesions.

## Figures and Tables

**Table 2:** IVUS and OCT for the imaging of calcified lesions.

	IVUS	OCT
Resolution	Conventional IVUS 100 μm, HD-IVUS up to 22 μm	High, 10–20 μm
Calcium detection	Very high sensitivity and specificity	High sensitivity and specificity
Calcium appearance	Bright hyperechoic signal (echodense) with posterior ‘’acoustic shadow’’	Low signal intensity areas with well defined borders
Microcalcifications	Not detected	Detected
Superficial calcium	Detected	Detected
Deep calcium	Detected but can be hidden from superficial calcium	Limited deep calcium localization
Calcium thickness	Cannot be measured directly (acoustic shadowing)	Can be measured
Calcium arc	Can be measured	Can be measured
Calcium length	Can be measured	Can be measured
Calcium area volume	Cannot be measured	Can be measured
Ostial lesions	Can be assessed	Cannot be assessed
Calcified nodules	Can be identified	Can be identified
Firocalcific plaque	Possible assessment	Difficult assessment
Assessment of calcium modification technique’s effect (fractures, fissures)	Possible	Possible
Stent guidance lesion length, vessel diameter, landing zone	Feasible	Feasible
Estimation of calcium in large caliber/aneurysmatic vessels	Feasible	Difficult/not possible (low penetration depth)
Estimation of calcium in aortic-ostial lesions	Feasible	Difficult
Identification of complications dissection, hematoma, thrombus stent underexpansion/malapposition	Feasible	Feasible Better for malapposition assessment
Criteria of calcium severity and risk of stent underexpansion indicating the need for lesion modification	Superficial calcium angle of >270° (longer than 5 mm) 360° angle of superficial calcium Calcified nodule Vessel diameter ≤ 3.5 mm	Calcium max angle > 180° Calcium max thickness > 0.5 mm Calcium length > 5 mm

Abbreviations: IVUS: IntraVascular UltraSound; HD: High Definition; OCT: Optical Coherence Tomography.

## Data Availability

No new data are created.
